# A retrospective analysis of malaria deaths in the pre- and intra- COVID 19 pandemic era, Ghana, 2016–2021

**DOI:** 10.1371/journal.pone.0286212

**Published:** 2024-02-06

**Authors:** Keziah L. Malm, Nana Yaw Peprah, Wahjib Mohammed, Boakye-Yiadom Adomako, Samuel Oppong, Paul Boateng, George Asumah Adu, Dora Dadzie, Grace Adza, Ivy Forson, James Frimpong

**Affiliations:** 1 National Malaria Control Programme, Public Health Division, Ghana Health Service, Accra, Ghana; 2 Department of Public Health, Cape Coast Teaching Hospital, Cape Coast, Ghana; University of Health and Allied Sciences, GHANA

## Abstract

**Background:**

Global efforts over the years have resulted in a 27% reduction in malaria incidence and an estimated 51% reduction in malaria mortality since 2000. Meanwhile, COVID-19 pandemic disrupted provision and utilization of malaria services, leading to a surge in malaria incidence and mortality. Globally, 627000 malaria deaths were recorded in 2020, representing about 69000 more deaths compared to 2019. Also, 14 million more cases of malaria were recorded in 2020 compared to 2019. This study sought to determine whether excess malaria deaths were recorded in Ghana during the COVID-19 pandemic era.

**Methods:**

This was a descriptive study on routine malaria mortality data in Ghana for the period 2016 to 2021. Data was retrieved from the District Health Information Management System using a data extraction guide. Excess mortality was defined as occurrence of malaria deaths more than expected value for the period 2020 and 2021. The expected number of mortalities for 2020 and 2021 were determined using 2016 to 2019 average. Excess mortality (P-score) was estimated using the formula: [(reported mortalities–expected mortalities)/expected mortalities X 100%]. Data were summarized and processed in Microsoft excel version 16.0. Malaria mortality in Ghana and its regions was described using tables and line graphs.

**Results:**

An average of 535 malaria deaths per year were recorded nationwide from 2016 to 2020. About 50% (1603/3207) of deaths occurred in children aged less than five years. The p-scores for the country were -53% and -58% for 2020 and 2021 respectively. No region recorded excess all-age malaria mortality in 2020, rather significant reduction. Stratified by age, Greater Accra region reported 90% higher than expected deaths among persons aged five years and above in 2020 (p-score = 90%, 95% CI: 21–159). All regions reported reduction in under-five mortality in 2020. No significant excess malaria mortalities were reported among the regions in 2021.

**Conclusion:**

Although negative p-scores suggested a decline in malaria mortalities nationwide, some regions recorded excess deaths during the COVID-19 pandemic era. There is a need to integrate COVID-19 control activities with malaria control and prevention efforts to mitigate the impact of COVID-19 on malaria case management and mortality.

## Background

The COVID-19 pandemic began in December 2019, disrupting healthcare systems and global health security. The novel coronavirus was first discovered in the Wuhan city of China in 2019 [1]. Owing to the rapid rate of spread of the virus across borders, it was declared a pandemic on March 11^th^, 2020 by the World Health Organization. As of November 27, 2022, COVID-19 had affected at least 637 million people and killed about 6.6 million globally [1]. Travel restrictions, curfews, social distancing, city lockdowns, quarantine, and isolation of affected individuals, were some protective measures outlined by the WHO to mitigate the pandemic. The high fatality rate of this virus, coupled with its devastating impact on economies affected access and utilization of vital health services, including malaria-related services [2]. Also, change in mode of delivery of services, staff workload, challenges in supply of commodities, stigma, and fear of infection, directly and indirectly affected the provision and use of essential health services [3].

Prior to COVID-19 pandemic, advancements were made towards the global control and elimination of malaria. Global efforts according to the WHO led to a 27% reduction in malaria incidence and an estimated 51% reduction in malaria mortality rate since 2000 [1]. However, the pandemic disrupted provision and utilization of malaria services, leading to a surge in malaria incidence and mortality [2]. Globally, 627000 malaria deaths were recorded in 2020, representing about 69000 more deaths compared to 2019. Also, 14 million more cases of malaria were recorded in 2020 compared to 2019 [2]. Sub-Saharan Africa accounted for 95% of all cases and 96% of all malaria deaths seen in 2020. Empirical studies have reported a surge in malaria mortality and morbidity in the COVID-19 era. In Zimbabwe, a study to assess malaria incidence and mortality during COVID-19 pandemic discovered an excess of 30, 000 malaria cases in 2020 compared to the same period in 2018 and 2019 [4]. Also, in the Northern region of Ghana, an increase in malaria incidence was noted among pregnant women in 2020 [5].

Over the years, through the National Malaria Control Programme (NMCP), Ghana has made a significant progress towards an ultimate goal of malaria elimination. Through a combination of intervention strategies, implementation of strategic plans and funding support, malaria morbidity and mortality has reduced over the years [6]. To further reduce malaria burden, a strategic plan was adopted to reduce malaria burden by 75% between 2015 to 2020 [6]. Use of Long-lasting Insecticide Net (LLIN), Seasonal Malaria Chemoprevention (SMC), Sulphadoxine Pyrimethamine for pregnant women, Indoor Residual Spraying (IRS), Larval Source Management and appropriate case management using Artemisinin-Based Combination Therapy (ACTs) are major interventions used by the NMCP for control and eventual elimination of malaria in Ghana.

In a highly malaria endemic country, the risk of retrogression in malaria control was anticipated due to the potential adverse effect of COVID-19 pandemic on health care delivery. In Ghana, the COVID-19 pandemic affected the delivery of malaria services including reduction in access to laboratory and treatment services, interruption/modification of LLINs, indoor residual spraying, and seasonal malaria chemoprevention campaigns among others. For example, while OPD attendance increased by 6.5% between 2018 & 2019, it reduced by 10.6% between 2019 and 2020. At the same time, the number of suspected malaria cases which increased by 8.7% between 2018 & 2019 and saw a reduction of 14.1% between 2019 & 2020. Inpatient services were similarly affected with 5.1% and 21.6% reductions in total admissions and malaria admissions respectively [7]. Moreover, there was a disruption in the supply of malaria commodities [8]. It was however unclear the extent to which COVID-19 affected the progress of malaria control in Ghana. It was against this background that the NMCP saw the need to conduct this study to assess the effects of COVID-19 on malaria mortality in Ghana [9]. This study sought to determine whether excess malaria deaths were recorded in Ghana during the COVID-19 pandemic era.

## Methods

### Study design

This study was a retrospective descriptive study which involved analysis of secondary data. Malaria mortality from January, 2016 to December, 2021 were retrieved from the Ghana Ministry of Health’s District Health Information Management Systems (DHIMS). Data on malaria mortality among children aged less than five years and individuals above five years of age were retrieved for all sixteen regions of Ghana. This was to enable characterization of malaria mortality by person, place and time before COVID-19 (2016 to 2019) and during COVID-19 period (2020 to 2021).

### Study setting

Ghana is a country located in Sub Saharan Africa and borders with Togo in the west, Burkina Faso in the North and Gulf of Guinea in the south. The country is made of 16 different regions which have been zoned into three; northern, middle and coastal zones. Ghana currently has a population of about 31 million with Greater Accra (17.7%), Ashanti (17.6%), Eastern (9.5%) and Central (9.3%) regions containing more than half of the total population [10]. Ghana currently has a 2.1% annual population growth rate and an average household size of 3.6 [10]. Ghana is a malaria endemic country with seasonal patterns that is strongest between July and November. The seasonal variations in malaria incidence are linked to the two major raining seasons experienced in the northern part of the country. Therefore, major malaria control interventions such as the Seasonal Malaria Chemoprevention is carried out during this period to protect children below five years of age against malaria. All health facilities in Ghana undertake malaria control efforts through out-patient sessions, antenatal care, child welfare clinic, immunization sessions and mass campaigns. These facilities carry out the following; testing and treatment for malaria, health education on malaria, and administration of sulphadoxine-pyrimethamine (SP) and long-lasting insecticide bed nets (LLIN) to pregnant women.

### Data source

Malaria mortality data for the study was extracted from the Ghana District Health Information Management System (DHIMS), a countrywide web-based database for collecting, collating, transmitting, analyzing, and storing routine health service data. DHIMS is a customized version of DHIS2, an open-source web-based platform commonly used as a health management information system (HMIS) across 76 lower and middle income countries across the world (https://dhis2.org/about/). Malaria death counts, aggregated on monthly bases, are keyed into DHIMS at health facility level by health records officers. Once entered, data is available in real time to persons with authorized access at levels of service delivery.

### Data collection

A data extraction guide was used to extract data on malaria mortality from the District Health Information System. All malaria deaths recorded in Ghana from January 1^st^, 2016 to December 31^st^, 2021 were retrieved from the DHIMS. A malaria death in this study was defined as the death of an inpatient malaria case which was ascribed to malaria., Aggregated monthly data on malaria deaths for all sixteen regions were retrieved from the DHIMS.

### Data analysis

Data retrieved from the DHIMS was processed and summarized using Microsoft Excel version 16.0. Monthly number of malaria deaths from January, 2016 to December, 2021 were aggregated for all sixteen regions to provide total number of deaths recorded across the period from 2016 to 2021. Further, for each region, data were grouped into two age groups: those below five years and those five years and above of age.

The cumulative number of deaths from 2016 to 2019 before COVID-19 era was compared to deaths recorded from 2020 to 2021 during COVID-19 era. Excess mortality during the COVID-19 era was defined as occurrence of malaria deaths in excess of value expected for the period 2020 and 2021. The expected number of mortalities for 2020 and 2021 were determined using 2016 to 2019 average. Excess mortality (P-score) was estimated using the formula: [(reported mortalities–expected mortalities)/expected mortalities X 100%], whereby reported mortalities is the number of deaths for 2020 and 2021 and expected mortality is the average number of mortalities for 2016 to 2019. Uncertainty in excess deaths/p-scores was calculated using the formula: (reported deaths ± (1.96 * √ (root reported deaths)). P-scores were also computed and compared among the regions as well as between deaths occurring in children aged less than five years and those five years and above.

### Ethical considerations

The analysis was conducted by the National Malaria Control Programme, the institution mandated for malaria control activities in Ghana as part of quality improvement measures. Only aggregated data were extracted for analysis. The nature of the study did not require ethical approval.

## Results

### Trend of malaria mortality in Ghana, 2016 to 2021

**Fig 1:**
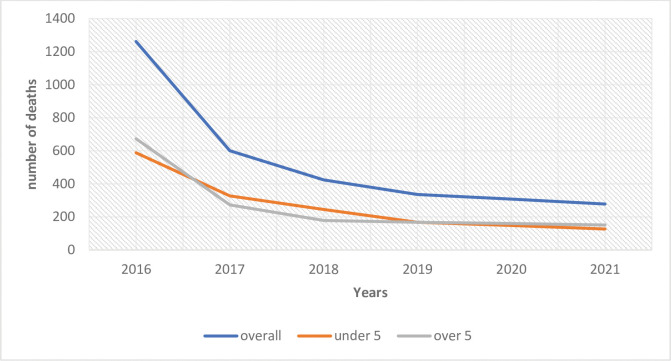
Trend of malaria mortality, Ghana, 2016 to 2021.

A total of 3,207 people died from malaria between 2016 and 2021. About 50% (1603/3207) of deaths occurred in children aged less than five years. In general, malaria mortality decreased across the years from 2016 (1,261) to 2021 (278) (Fig 1). In 2016, a total of 588 under-five children and 673 persons aged five or more years died from malaria. In 2021, however, the number of deaths were lower: 127 among the under-five and 151 for persons aged five or more years (Fig 1).

### Excess malaria mortality, 2020–2021

The reported mortality for all age malaria for 2020 was 308 against an expected value of 656. There were 53% (95% CI: -48 to -58%) fewer deaths in 2020 compared to the pre-COVID era. Similarly, no excess malaria deaths were found among the age-groups under-five and over-five. Rather, there were significant reductions. Under-five malaria mortalities were 55% (95% CI: -55 to -63%) lower whilst over-five malaria mortalities were 52% fewer (95% CI: -43 to -58%) (Table 1). In 2021, malaria mortalities were 58% (95% CI: -53 to -63%) lower than expected. Under-five (-62%) as well as over-five malaria mortalities (-53%) were also significantly lower (Table 1).

**Table 1:** Excess malaria mortality, Ghana, 2020–2021.

	Expected mortality*	Reported mortality (95% CI)	Excess deaths (95% CI)	P-score % (95% CI)
**2020**				
Total	656	308 (274 to 342)	-348 (-314 to—382)	-53% (-48 to -58)
Under-five malaria	332	148 (124 to 172)	-184 (-160 to -208)	-55% (-48 to -63)
Over 5 malaria	324	160 (135 to 185)	-164 (-139 to -189)	-51% (-43 to -58)
**2021**				
Total	656	278 (245 to 311)	-378 (-345 to -411)	-58% (-53 to -63)
Under-five malaria	332	127 (105 to 149)	-205 (-183 to -227)	-62% (-55 to -68)
Over-5 malaria	324	151 (127 to 175)	-173 (-149 to -197)	-53% (-46 to -61)

* Expected mortality figures based on 2016–2019 average

### Regional distribution of malaria mortality, 2016 to 2021

Overall, there were variations in trend of malaria mortality among regions. Some regions saw a surge in number of malaria deaths across the years while others recorded a decline in malaria mortality. For instance, in 2016, the Northern region recorded the highest number of malaria deaths of 236. However, this decreased significantly leading to 43 and 45 deaths in 2020 and 2021 respectively. Also, in the Oti and Savanna regions, malaria deaths decreased from 43 and 45 in 2016 to 7 and 11 respectively in 2020. Regions such as the Greater Accra region noted slight decrease in malaria deaths from 40 in 2016 to 33 in 2020. Meanwhile, in the Ahafo region, a surge was seen in malaria deaths from 2 in 2016 to 6 and 7 in 2020 and 2021 respectively (Table 2).

**Table 2:** Regional distribution of malaria deaths, Ghana, 2016 to 2021.

Regions	2016	2017	2018	2019	2020	2021
Ahafo	2	6	4	6	6	7
Ashanti	144	74	64	31	42	16
Bono	54	28	25	29	17	34
Bono East	65	52	41	34	14	31
Central	104	35	22	22	18	9
Eastern	62	41	38	41	29	29
Greater Accra	40	20	20	16	33	22
North East	71	52	16	15	14	14
Northern	236	127	84	52	43	45
Oti	43	5	12	11	7	7
Savannah	45	21	19	5	11	5
Upper East	102	33	13	7	1	6
Upper West	69	34	24	18	28	19
Volta	121	23	11	26	17	13
Western	66	41	22	14	14	11
Western North	37	8	9	9	14	10

### Excess malaria mortality by region, 2020–2021

No region recorded excess malaria mortality in 2020. Contrastingly, all recorded significant reduction in mortality. Ahafo region reported 2 excess deaths (p-score of 50%); however, this was not significant (95% CI: -70% to 170%) (Table 3). Stratified by age, Greater Accra region reported 90% higher than expected deaths among persons aged five years and above in 2020 (p-score = 90%, 95% CI: 21–159). All regions reported reduction in under-five mortality in 2020.

**Table 3:** Regional variation in excess malaria mortality, Ghana, 2020.

Regions	Expected mortality	Reported mortality (95% CI)	Excess deaths (95% CI)	P-score % (95% CI)
**Ahafo**				
Total	4	6 (1 to 11)	2 (-3 to 7))	50 (-17 to 100)
Under-five malaria	3	4 (0 to 8)	1 (-3 to 5))	45 (-97 to 188)
Over 5 malaria	1	2 (-1 to 5)	1 (-2 to 4))	60 (-162 to 282)
**Ashanti**				
Total	**77**	42(29 to 55)	-35(-47 to -22)	-45(-62 to -29)
Under-five malaria	27	16(8 to 24)	-11(-19 to -3)	-41(-70 to -12)
Over 5 malaria	50	26(16 to 36)	-24(-34 to -14)	-48(-68 to -28)
**Bono**				
Total	34	17(9 to 25)	-17(-25 to -19)	-50(-74 to -26)
Under-five malaria	15	10(4 to 16)	-5(-11 to 2)	-31(-74 to 12)
Over 5 malaria	20	7(2 to 12)	-13(-18 to -7)	-64(-91 to -38)
**Bono East**				
Total	48	14(7 to 21)	-34(-41 to -27)	-71(-86 to -56)
Under-five malaria	33	8(2 to 14)	-25(-31 to -20)	-76(-96 to -59)
Over 5 malaria	15	6(1 to 11)	-9(-14 to -4)	-59(-92 to -27)
**Central**				
Total	46	18(10 to 26)	-28(-36 to -19)	-61(-79 to -42)
Under-five malaria	18	9(3 to 15)	-9(-15 to -3)	-50(-83 to -17)
Over 5 malaria	28	9(3 to 15)	-19(-25 to -13)	-68(-89 to -46)
**Eastern**				
Total	46	29(18 to 40)	-17(-27 to -6)	-37(-60 to -14)
Under-five malaria	20	10(4 to 16)	-10(-16 to -3)	-49(-81 to -17)
Over 5 malaria	26	19(10 to 28)	-7(-16 to 1)	-28(-60 to 5)
**Greater Accra**				
Total	24	33(22 to 44)	9(-2 to 20)	38(-9 to 84)
Under-five malaria	9	4(0 to 8)	-5(-9 to -1)	-54(-99 to -9)
Over 5 malaria	15	29(18 to 40)	14(3 to 24)	90(21 to 159)
**North East**	**39**	**14(7 to 21)**	**-25(-32 to -18)**	**-64(-83 to -45)**
Total	26	9(3 to 15)	-17(-23 to -11)	-65(-88 to -43)
Under-five malaria	13	5(1 to 9)	-8(-12 to -4)	-62(-95 to -28)
Over 5 malaria			0	
**Northern**				
Total	125	43(30 to 56)	-82(-95 to -69)	-66(-76 to -55)
Under-five malaria	78	25(15 to 35)	-53(-63 to -43)	-68(-80 to -55)
Over 5 malaria	47	18(10 t0 26)	-29(-37 to -21)	-62(-79 to -44)
**Oti**				
Total	18	7(2 to 12)	-11(-16 to -6)	-61(-90 to -31)
Under-five malaria	8	4(0 to 8)	-4(-8 to 0)	-52(-99 to -4)
Over 5 malaria	10	3(0 to 6)	-7(-10 to -3)	-68(-104 to -33)
**Savannah**				
Total	23	11(4 to 18)	-12(-18 to -5)	-52(-80 to -23)
Under-five malaria	12	8(2 to 14)	-4(-10 to 1)	-35(-80 to 11)
Over 5 malaria	11	3(0 to 6)	-8(-11 to -4)	-71(-104 to -39)
**Upper East**				
Total	39	1(-1 to 3)	-38(-39 to -36)	-97(-102 to -92)
Under-five malaria	16	0(0 to 0)	-16(-16 to -16)	-100(-100 to -100)
Over 5 malaria	23	1(-1 to 3)	-22(-23 to -20)	-96(-104 to -87)
**Upper West**				
Total	36	28(18 to 38)	-8(-19 to 2)	-23(-51 to 6)
Under-five malaria	20	19(10 to 28)	-1(-10 to 7)	-6(-48 to 36)
Over 5 malaria	16	9(3 to 15)	-7(-13 to -1)	-44(-81 to -7)
**Volta**				
Total	45	17(3 to 15)		
Under-five malaria	16	9	-7(-13 to -1)	-43(-80 to -6)
Over 5 malaria	30	8(2 to 14)	-22(-27 to -16)	-73(-92 to -54)
**Western**				
Total	36	14(7 to 21)	-22(-29 to -14)	-61(-81 to -40)
Under-five malaria	23	5(1 to 9)	-18(-22 to -13)	-78(-97 to -59)
Over 5 malaria	13	9(3 to 15)	-4(-10 to 2)	-31(-76 to 14)
**Western North**				
Total	16	14(7 to 21)	-2(-9 to 6)	-11(-58 to 35)
Under-five malaria	10	8(2 to 14)	-2(-7 to 4)	-18(-75 to 39)
Over 5 malaria	6	6(1 to 11)	0(-5 to 5)	0(-80 to 80)

No significant excess malaria mortalities were reported among the regions in 2021 when compared to the pre-COVID-19 era (Table 4). Although Ahafo, Bono East and Greater Accra had more deaths than expected among the age group five years and over, these excesses were not significant.

**Table 4:** Regional variation in excess malaria mortality, Ghana, 2021.

Regions	Expected mortality	Reported mortality (95% CI)	Excess deaths (95% CI)	P-score % (95% CI)
**Ahafo**				
Total	4	7 (2 to 12)	3 (-2 to 8)	75 (-55 to 205)
Under-five malaria	3	3 (0 to 6)	0 (-3 to 4)	9 (-144 to 133)
Over 5 malaria	1	4 (0 to 8)	3 (-1 to 7)	220 (-94 to 534)
**Ashanti**				
Total	77	16(8 to 24)	-61(-69 to -53)	-79(-89 to -69)
Under-five malaria	27	7(2 to 12)	-20(-25 to -15)	-74(-93 to -55)
Over 5 malaria	50	9(3 to 15)	-41(-47 to -35)	-82(-94 to -70)
**Bono**				
Total	34	34(23 to 45)	0(-11 to 11)	0(-34 to 34)
Under-five malaria	15	12(5 to19)	-3(-9 to 4)	-17(-64 to 30)
Over 5 malaria	20	22(13 to 31)	3(-7 to 12)	13(-34 to 60)
**Bono East**				
Total	48	31(20 to 42)	-17(-28 to -6)	-35(-58 to -13)
Under-five malaria	33	14(7 to 21)	-19(-27 to -12)	-58(-80 to -36)
Over 5 malaria	15	17(9 to 25)	2(-6 to 10)	15(-40 to 70)
**Central**				
Total	46	9(3 to 15)	-37(-43 to -31)	-80(-93 to -67)
Under-five malaria	18	4(0 to 8)	-14(-18 to -10)	-78(-100 to -56)
Over 5 malaria	28	5(1 to 9)	-23(-27 to -18)	-82(-98 to -66)
**Eastern**				
Total	46	29(18 to 40)	-17(-27 to -6)	-37(-60 to -14)
Under-five malaria	20	8(2 to 14)	-12(-17 to -6)	-59(-87 to -31)
Over 5 malaria	26	21(12 to 30)	-5(-14 to 4)	-20(-54 to 14)
**Greater Accra**				
Total	24	22(13 to 31)	-2(-11 to 7)	-8(-47 to 30)
Under-five malaria	9	6(1 to 11)	-3(-8 to 2)	-31(-86 to 23)
Over 5 malaria	15	16(8 to 24)	1(-7 to 9)	5(-46 to 56)
**North East**	**39**	**14(7 to 21)**	**-25(-32 to -18)**	**-64(-83 to -45)**
Total	26	8(2 to 14)	-18(-24 to -12)	-69(-91 to -48)
Under-five malaria	13	6(1 to 11)	-7(-12 to -2)	-54(-91 to -17)
Over 5 malaria			0	
**Northern**				
Total	125	45(32 to 58)	-80(-93 to -67)	-64(-74 to -53)
Under-five malaria	78	28(18 to 38)	-50(-60 to -39)	-64(-77 to -51)
Over 5 malaria	47	17(9 to 25)	-30(-38 to -22)	-64(-81 to -47)
**Oti**				
Total	18	7(2 to 12)	-11(-16 to -6)	-61(-90 to -31)
Under-five malaria	8	4(0 to 8)	-4(-8 to 0)	-52(-99 to -4)
Over 5 malaria	10	3(0 to 6)	-7(-10 to -3)	-68(-104 to -33)
**Savannah**				
Total	23	5(1 to 9)	-18(-22 to -13)	-78(-97 to -59)
Under-five malaria	12	2(-1 to 5)	-10(-13 to -7)	-84(-106 to -61)
Over 5 malaria	11	3(0 to 6)	-8(-11 to -4)	-71(-104 to -39)
**Upper East**				
Total	39	6(1 to 11)	-33(-37 to -28)	-84(-97 to -72)
Under-five malaria	16	1(-1 to 3)	-15(-17 to -13)	-94(-106 to -82)
Over 5 malaria	23	5(1 to 9)	-18(-22 to -13)	-78(-97 to -58)
**Upper West**				
Total	36	19(10 to 28)	-17(-26 to -9)	-48(-71 to -24)
Under-five malaria	20	11(4 to 18)	-9(-16 to -3)	-46(-78 to -14)
Over 5 malaria	16	8(2 to 14)	-8(-14 to -2)	-50(-85 to -15)
**Volta**				
Total	45	12(5 to 19)	-33(-40 to -26)	-73(-88 to -58)
Under-five malaria	16	10(4 to 16)	-6(-12 to 0)	-37(-76 to 3)
Over 5 malaria	30	2(-1 to 5)	-28(-30 to -25)	-93(-103 to -84)
**Western**				
Total	36	11(4 to 18)	-25(-31 to -18)	-69(-87 to -51)
Under-five malaria	23	5(1 to 9)	-18(-22 to -13)	-78(-97 to = 59)
Over 5 malaria	13	6(1 to 11)	-7(-12 to -2)	-54(-91 to -17)
**Western North**				
Total	16	10(4 to 16)	-6(-12 to 0)	-37(-76 to 3)
Under-five malaria	10	4(0 to 8)	-6(-10 to -2)	-59(-99 to -19)
Over 5 malaria	6	6(1 to 11)	0(-5 to 5)	0(-80 to 80)

## Discussion

COVID-19 pandemic impacted various aspects of Ghana’s health care system including the malaria control efforts and interventions [2]. Hence, this study sought to compare number of malaria deaths recorded pre- and intra- COVID 19 pandemic era to inform decision making. Findings from the study showed that, malaria mortality in Ghana declined in both children less than five years and those above five years from 2016 to 2021. According to study findings, fewer number of deaths were recorded among children less than five years in 2020 and 2021 as compared to 2019. Similarly, among those over five years, fewer number of malaria deaths were recorded in 2020 and 2021 compared to 2019. In line with these findings, a scoping review conducted to ascertain COVID-19 impact on Sub Saharan Africa reported a decline in malaria cases and deaths in high malaria endemic countries in Sub Saharan Africa [11]. Contrary to this finding, other empirical studies have found increased malaria mortality during COVID-19 era. For instance, a report from WHO indicated a 12% surge in malaria deaths from 2019 to 2020 [2]. In addition, a study carried out in Africa projected about 81,000 additional malaria deaths in Nigeria during COVID-19 era [9]. Several factors could have contributed to the reduction in malaria deaths as reported in this study. For instance, this could be due to the reduced hospital attendance during the COVID-19 pandemic. It could also, be attributable to the COVID-19 mitigation strategies outlines by the National Malaria Control Programme in the era of COVID-19, notably procurement and timely supply of PPEs and malaria commodities, using non-contact strategies to perpetrate malaria BCC messages, particularly on health seeking behavior, frequent home visitation by community health workers for early identification and referral of the sick among others. Malaria control activities such as IPT for pregnant women, routine distribution of LLINs and other malaria management policies have over the years been integrated into routine health services, thereby, reducing the impact of pandemics on malaria control. Moreover, other interventions such as the seasonal malaria chemoprevention and LLIN campaigns continued during the pandemic era with strict adherence to COVID-19 prevention protocols. Secondly, the reduction in malaria mortality as found in this study could be due to under reporting resulting from shift in focus from other diseases towards the mitigation of COVID-19 by health facilities and health workers [12]. Moreover, malaria and COVID-19 shared similar presentation and symptoms including fever, tiredness, headache and breathing difficulties [8]. Hence, this could have led to misdiagnosis of malaria for COVID-19 particularly in situations where health providers depended solely on signs and symptoms presented by patients [8]. Also, patients with malaria and COVID-19 as co-morbidities were likely to receive the latter as diagnosis since it was more popular and had inordinate attention than other diseases in 2020 and 2021. Though, malaria deaths reduced country wide in Ghana during COVID-19 era, excess malaria deaths were recorded in the Greater Accra region during this period. In general, COVID-19 pandemic affected access and delivery of primary health services [13]. It further influenced the management of malaria cases due to the fear of patients to visit health facilities [13]. This could be the reason for the increased malaria deaths recorded in this region. Additionally, the Greater Accra region was noted as Ghana’s hot spot of COVID-19 during the first few months after the virus was confirmed in the country. This resulted in health facilities becoming overwhelmed and shifted focus from other tropical diseases to the control and management of COVID-19 cases [12]. This shift in focus by health providers could be the cause of increased malaria deaths during the pandemic era compared to pre-pandemic era in the Greater Accra region. Hence, there is a need to integrate COVID-19 preventive and control measures with existing malaria control and prevention interventions to reduce the impact of COVID-19 on malaria mortality and morbidity.

### Study limitations

This study utilized data from routine surveillance report on malaria. In calculating excess deaths, we assumed the reporting completeness for malaria deaths to be high throughout the review period. However, the surveillance system could have been affected by the COVID-19 pandemic, leading to erratic reporting by health facilities. Uncertainty of reported deaths during the pandemic was thus accounted for by computing 95% confidence intervals. Also, the average number of deaths for the period 2016 to 2019 was used to estimate the expected number of deaths for 2020 and 2021. Because this method does not account for the year-to-year variation in trends, we may have misestimated the magnitude of excess deaths. This study did not take into consideration people who died at home and failed to report to health facilities.

### Conclusion and recommendations

Overall, fewer malaria deaths were recorded in Ghana during the COVID-19 pandemic era as compared to the pre-covid-19 era. At the regional level, only the Greater Accra region recorded excess malaria deaths in the COVID-19 pandemic era. With the Greater Accra being Ghana’s capital, this observation could be attributed to the reason that, the region was the hotspot for the pandemic. Though, these findings mean that, the Ghana’s malaria control programme was not greatly impacted by the pandemic, there is a need to maintain a resilient health care system in Ghana which is able to swiftly adjust to infectious disease outbreaks. For the Greater Accra region, there is a need to double malaria control efforts to be able to meet targets. Moreover, there is a need for stakeholders in the health sector to strategize towards integrating COVID-19 control efforts with other malaria control interventions at the regional and district levels to reduce the impact of COVID-19 on malaria control and prevention. However, a community-based study using qualitative approach is needed to further estimate the impact of COVID-19 on the delivery and access to malaria related services.

## Supporting information

S1 Table (XLSX)
